# Effects of two types of activated carbon on the properties of vegetation concrete and *Cynodon dactylon* growth

**DOI:** 10.1038/s41598-020-71440-w

**Published:** 2020-09-02

**Authors:** Jiazhen Gao, Daxiang Liu, Yakun Xu, Jiangang Chen, Yueshu Yang, Dong Xia, Yu Ding, Wennian Xu

**Affiliations:** 1 Engineering Research Center of Eco-Environment in Three Gorges Reservoir Region (China Three Gorges University), Ministry of Education, Yichang, 443002 China; 2 Key Laboratory of Mountain Hazards and Surface Processes, Institute of Mountain Hazards and Environment, Chinese Academy of Sciences, Chengdu, 610041 China; 3 Key Laboratory of Geological Hazards on Three Gorges Reservoir Area (China Three Gorges University), Ministry of Education, Yichang, 443002 China; 4 Key Laboratory of Disaster Prevention and Mitigation (China Three Gorges University), Hubei Province, Yichang, 443002 China

**Keywords:** Environmental sciences, Engineering

## Abstract

Vegetation concrete is one of the most widely used substrates for slope ecological protection in China. However, there are still some imperfections that are disadvantageous for plant growth, such as high density, low porosity, insufficient nutrient retention ability and so on. In this paper, the effect of wood activated carbon and mineral activated carbon on the physicochemical properties of vegetation concrete is studied. The experimental results show that the activated carbon proportion in vegetation concrete is positively related to the porosity, permeability coefficient, water holding capacity, and nutrient content and retention ability, while it is negatively related to the dry density, water retention ability, cohesive force and internal friction angle. However, it should be noticed that when the proportion exceeds 2%, the average height, aboveground biomass and underground biomass of *Cynodon dactylon* decrease with increasing proportion of activated carbon. The effect of wood activated carbon is generally more remarkable than that of mineral activated carbon. In addition, according to the research results, the effect of activated carbon on vegetation concrete can last for at least half a year, although it does slowly deteriorate with increasing time. By comprehensive consideration of the current industry standard, previous research results and economical reasoning, the recommended type of activated carbon is wood, with a corresponding suitable proportion ranging between 1 and 2%.

## Introduction

Vegetation concrete ecological protection technology, the theory of which refers to both civil engineering and ecology fields, is one of the most widely used technologies for bare steep slopes in China^1^. Generally, vegetation concrete is composed of soil, cement, organic material, microbial agent, plant seeds and water. The proportion of each component is mainly determined by the regional climate, geological condition, slope angle, and the soil texture of the engineering location^2,3^. Through ejection onto a slope by an air compressor machine, vegetation concrete has the function of revegetation and shallow protection for bare slopes. Currently, the technology has been used in more than twenty provinces in China and has formed a national industry standard in 2016^4^. According to experience from numerous engineering studies, soil composed of an approximate content of sand and clay content is the best soil type for the preparation of vegetation concrete. However, the parent material for soil around a bare slope always features some weathering products of rock, and the sand content is obviously higher than the clay content. Consequently, due to the superposed hydration effect of cement, vegetation concrete can be easily hardened, which is disadvantageous for plant growth and engineering performance.

Activated carbon that is carbonized and activated by coal, mineral, wood or some other raw materials at a high temperature exhibits features of numerous micropore structures, stable performance and non-pollution^5–7^. In recent years, activated carbon has attracted wide attention in the field of soil improvement. Some studies have pointed out that when activated carbon is mixed into soil, the density of the soil can be decreased and the porosity and field water capacity can be increased^8–10^. These results certify that activated carbon can reduce the degree of density and improve soil structure effectively. On the other hand, a certain degree of nutrient exists in activated carbon, which can enhance soil fertility and promote plant growth^11–14^. Moreover, large quantities of negative electrical charges are present on the surface of activated carbon, and many mineral elements can be adsorbed, which means that activated carbon can enhance the cation exchange level of the soil and retain nutrients; thus, the ability for continuous nutrient supply can be strengthened^15^. However, some studies have also indicated that an increased proportion of activated carbon in soil is not necessarily better, as too much activated carbon can obviously decrease the mechanical strength, leading to more serious water loss or soil erosion problems^16^. In addition, it is necessary to note that the performance effect can certainly depend on the parent material of the activated carbon, carbonized temperature, texture and granulometric composition of the soil and some other factors^17^. In addition, soluble carbon, transformed from solid state activated carbon through ageing, can easily run off due to flowing water^18^. Therefore, issues related to the suitable type, proportion and durability of activated carbon used in different types of soil are of concern in the field of soil improvement.

It is generally known that soil is the main component of ecological protection substrates. However, almost no study is found at present for activated carbon use in the field of slope ecological protection. Thus, to research optimization approaches for vegetation concrete ecological protection technology, it is significant to introduce activated carbon into normal vegetation concrete. In this paper, the effects of two typical types of activated carbon, wood and mineral activated carbon^19,20^, on the physicochemical properties of vegetation concrete were studied through an outdoor pot experiment. *Cynodon Dactylon*, as one of most commonly used plant in slope ecological protection, was chosen to research the effect of activated carbon on plant growth. Overall, the target of this research is to recommend a suitable type and proportion of activated carbon for use in vegetation concrete.

## Materials and methods

### Overview of the research area

The test site is located next to the Dixue building of the China Three Gorges University in Yichang city, Hubei Province, China (111°18′23″E, 30°43′06″N). This area belongs to the subtropical monsoon humid region. The annual average temperature and rainfall for this region is 16.9℃ and 1,215.6 mm, respectively. The rainfall mostly occurs during the months from July to September. The average annual frost-free period is 272 days, and the total annual sunshine hours are 1,100–1,300 h.

### Test materials

**Figure 1:**
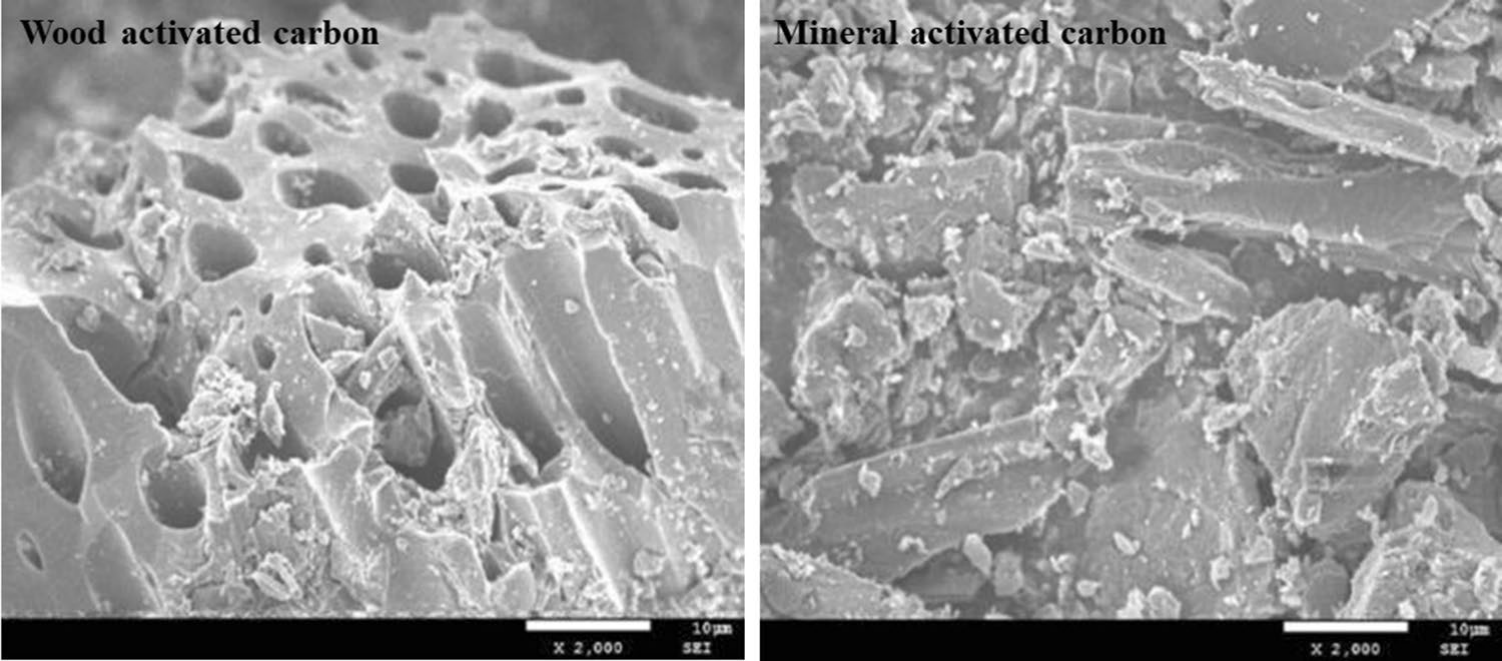
SEM images of the wood activated carbon (**A**) and mineral activated carbon (**B**) used in the experiment.

The main materials involved in this study were planting soil, cement, organic material, microbial agent, *Cynodon dactylon* plant seeds, wood activated carbon, mineral activated carbon and water. Planting soil was selected from natural yellow brown loam soil within 1 m of the surface depth at the test site. The soil was retrieved, dried, mashed, and sieved through a 2 mm sieve. The basic properties of the soil are shown in Table 1. The P.O 42.5 ordinary Portland cement used was produced by Huaxin Cement Co., LTD. The organic material, which was prepared from local fir sawdust, was dried in an oven first, and then sieved through a 2 mm sieve. The microbial agent used was a patented product provided by China Three Gorges University, which is rich in microorganisms such as nitrogen-fixing microorganisms, phosphorus-solubilizing microorganisms and potassium-solubilizing microorganisms. The wood and mineral activated carbon, pyrolyzed by high-quality wood chips and anthracite under anoxic conditions at 500 °C, respectively, were modified by FeCl_3_ and purchased from Runzhi Industry and Trade Co., Ltd. The particle sizes for the two types of activated carbon were both approximately 74 μm, and the specific physical and chemical properties are shown in Table 2. Moreover, the scanning electron microscope (SEM) images of the two types of activated carbon are shown in Fig. 1. It can be found that mineral activated carbon is a dense, long, strip-shaped solid sheet structure with a lower interparticle pore space, while wood activated carbon is a cylindrical structure with a more abundant pore structure and a larger specific surface area.*Cynodon dactylon* is a kind of warm-season perennial grass and widely used in slope ecological protection. Thus, the unshelled seed of *Cynodon dactylon* which was provided by Lantian Seeds Co., Ltd. was chosen in the experiment that began in summer. The weight ratios for each component of the vegetation concrete are expressed in Table 3. The usage amount for the seeds depends on the area of ecological restoration and is explained in the test design part.

**Table 1:** Basic properties of planting soil.

Planting soil type	Dry density/ (g/cm^3^)	pH	Particle size distribution /(%)			
			2–0.5 mm	0.5–0.25 mm	0.25–0.075 mm	<0.075 mm
Fine-grained sand soil	1.24	6.4	64.91	15.69	9.53	8.86

**Table 2:** Basic physicochemical properties of two types of activated carbon.

Property index	Available nitrogen/(mg/kg)	Available potassium/(mg/kg)	Available phosphorus/(mg/kg)	Stacking density/(g/cm^3^)	Carbon content/(%)	Ash content/(%)	Specific surface area/(m^2^/g)	CEC (cmol/kg)	pH
Wood activated carbon	151.21	175.73	97.48	0.54	71.9	15.8	78.11	12.31	8.31
Mineral activated carbon	135.18	112.44	69.12	0.62	62.7	20.7	23.36	22.12	9.37

**Table 3:** Weight ratio of each component in vegetation concrete.

Planting soil	Cement (P.O 42.5)	Organic material	Microbial agent	Water	Activated carbon
100	8	5	4	25	0/0.5/1/2/4/6

### Experimental design and sample collection

Six gradients were set for the content of the two types of activated carbon. Wood activated carbon (W) and mineral activated carbon (M) content accounted for 0%, 0.5%, 1%, 2%, 4% and 6% of the dry weight of the planting soil, respectively.

**Figure 2:**
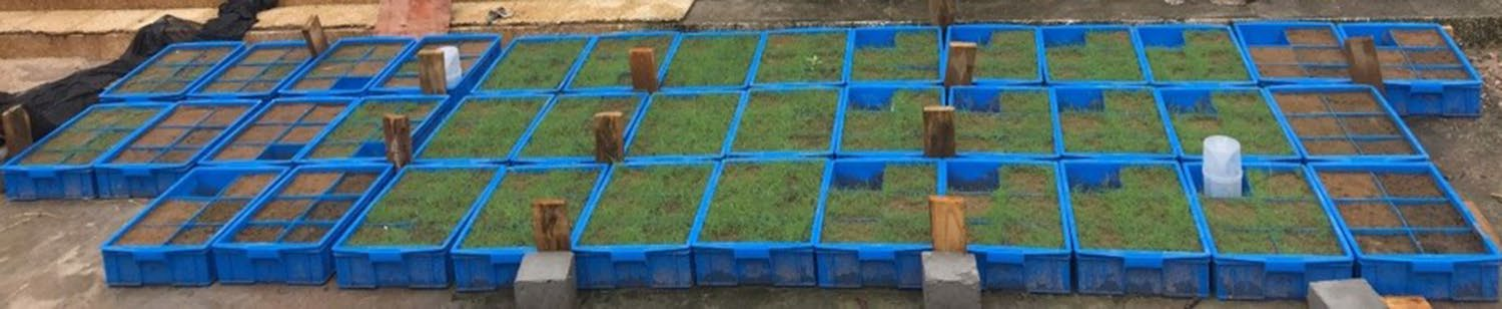
Planter arrangement in the experiment.

An outdoor pot experiment using a plastic planter was carried out, and each plastic planter used in the test was divided into six smaller planters according to the six different values of activated carbon content. The length, width and height of each smaller planter were 19.5 cm, 17 cm and 14 cm, respectively. Then, 4 kg of vegetation concrete was filled into each smaller planter. The sowing amount of *Cynodon dactylon* seeds was 20 g/m^2^ in accordance with existing engineering experience. The experiment began on June 1, 2018, and the first sampling started at 7 d to ensure that the hydration reaction of the cement was mostly complete. Then, samples were taken at 37 d, 97 d, and 187 d, successively. With regard to the physicochemical properties, 3 parallel samples were arranged for each treatment, and 144 small planters were needed. With regard to the shear strength, 48 small planters were needed. The biomass of the *Cynodon dactylon* was just measured at 187 d; thus, 36 small planters were needed. Therefore, 228 small planters were needed in total. Figure 2 shows the planter arrangement used in the experiment.

**Figure 3:**
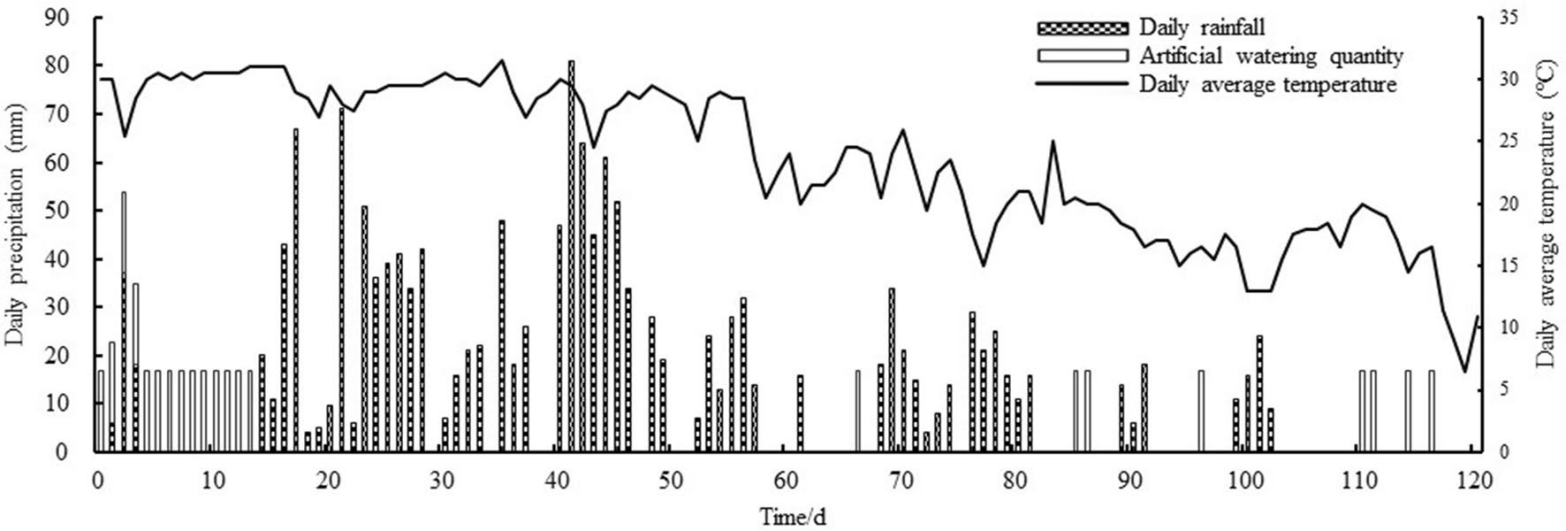
Artificial watering quantity, daily rainfall and average temperature during the experiment period.

During the experimental period, the daily rainfall, artificial watering quantity and average temperature were recorded, as shown in Fig. 3.

### Test methods

The dry density and porosity were measured by an oven drying method and drainage method, respectively^21^. The permeability coefficient was measured by a variable water level method^22^. The water holding capacity was represented by the saturated water content. With regard to the water retention ability, saturated samples were placed into a drying oven set to a temperature of 35 °C and weighed every 24 h for one week; then, the water retention ability was calculated using the water evaporation rate (*R*_*1*_) as follows:

1 $$R_{1} = \frac{{W_{c} }}{{W_{t} }}$$

where *W*_*c*_ is the cumulative evaporation weight of water at any time (g); *W*_*t*_ is the total weight of water in a saturated sample (g).

Samples of microgram quantities were observed by using a cold field emission scanning electron microscope. The cohesive force and internal friction angle were measured by a strain-controlled shear apparatus; the rate of shearing was 1.2 mm/min.

For the nutrient content, a SKALAR SAN + continuous flow analyser was used to measure the content of nitrate nitrogen (NO_3_^−^–N), ammonium nitrogen (NH_4_^+^–N), available phosphorus (PO_4_^3−^–P), available potassium (K^+^), respectively. In addition, the nutrient retention capability of the vegetation concrete was represented by the nutrient leaching rate as measured by a pillar leaching test, in which the sample dimensions were *Φ* 50.46 mm × 100 mm. The nutrient leaching rate (*R*_*2*_) can be calculated as follows:

2 $$R_{2} = \frac{{N_{l} }}{{N_{s} }}$$

where *N*_*l*_ is the nutrient content in the leachate (mg); *N*_*s*_ is the nutrient content in the sample before being leached (mg).

The average plant height for the 25 tallest plants selected from each small planter was measured by a ruler at 7 d, 37 d, 97 d, and 187 d, respectively. All plants were harvested and dried at 187 d; thus, the aboveground and underground biomass for *Cynodon dactylon* was obtained.

## Results

### Change laws for the dry density and porosity

**Figure 4:**
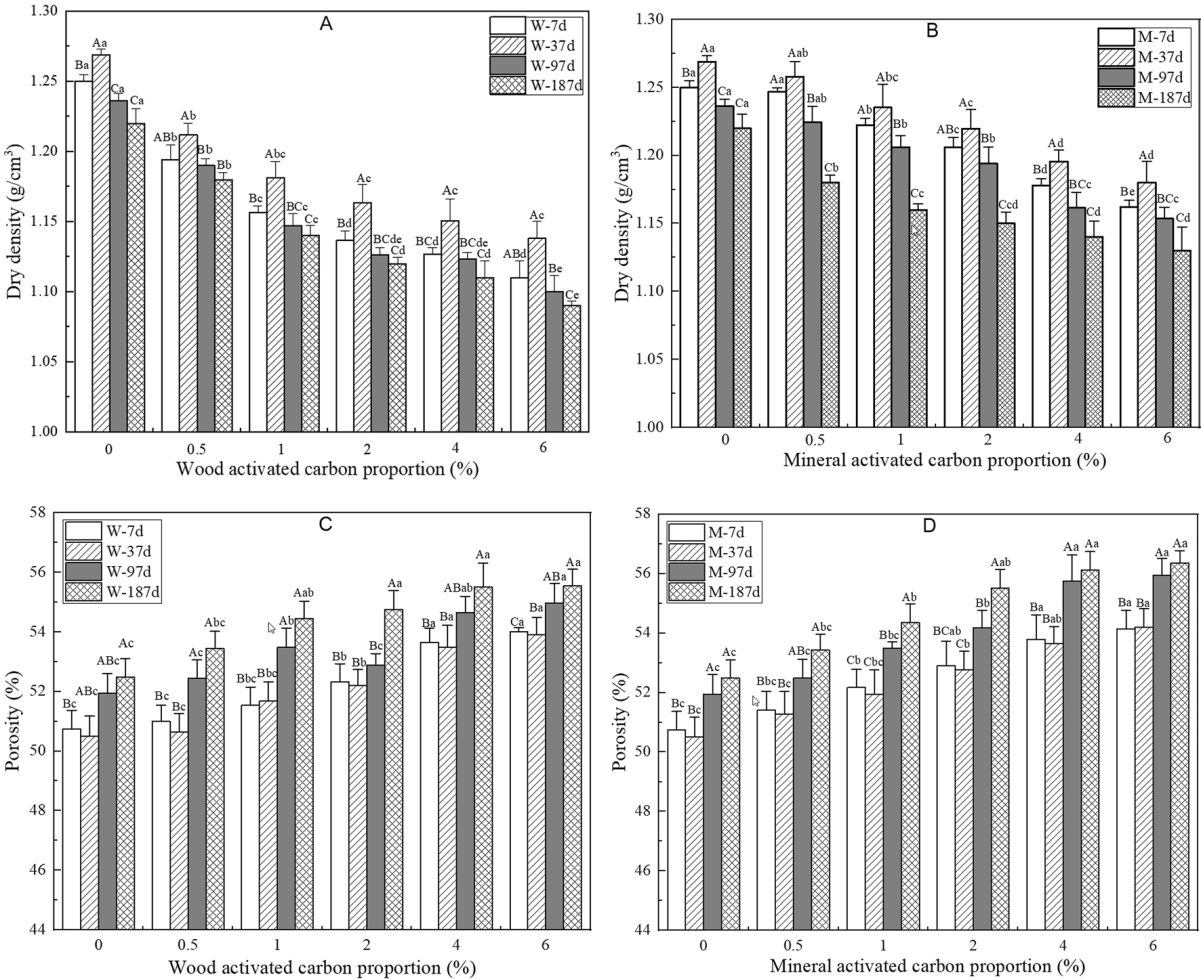
Effects of activated carbon on the dry density (**A,B**) and porosity (**C,D**) of vegetation concrete. Different capital letters indicate significant differences between different times (*P* < 0.05). Different small letters indicate significant differences between different activated carbon proportions (*P* < 0.05).

Density and porosity are the most important physical properties of vegetation concrete for plant growth^23^. As shown in Fig. 4A, B, the dry density of vegetation concrete decreases and the porosity increases significantly with increasing proportion of activated carbon. As time goes on, the dry density increases first from 0 to 37 d and then decreases after 37 d. The trend for porosity shows the opposite behaviour according to Fig. 4C, D. The reason for this trend may be that the main consolidation of the vegetation concrete occurred and the effect of plant roots on vegetation concrete was slight before 37 d. With plant growth, pores in the vegetation concrete increased constantly; thus, the dry density decreased. From Fig. 4, it can also be found that the dry densities for samples containing mineral activated carbon are higher than those for samples containing wood activated carbon. This finding may be due to the density of the mineral activated carbon being higher than that of the wood activated carbon.

### Change law for the permeability coefficient

**Figure 5:**
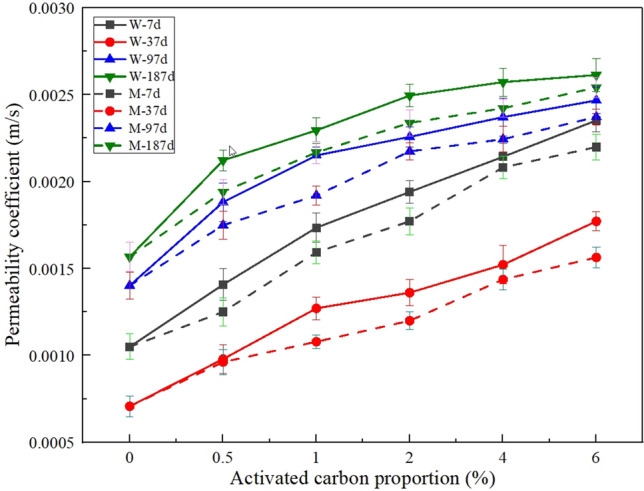
Effect of activated carbon on the permeability coefficient of vegetation concrete.

The curves for the permeability coefficient versus activated carbon proportion are shown in Fig. 5. Similar to porosity, the permeability coefficient increases with increasing proportion of activated carbon. Although the permeability coefficient at 37 d is lower than that at 7 d, it increases gradually from 37 to 187 d. And the permeability coefficients for samples containing wood activated carbon are generally higher than those for samples containing wood activated carbon. The reason for this may be wood activated carbon has a more abundant micropore structure.. Because an increase in the number of pores is advantageous for the formation of water flow channels^24^, the permeability coefficient is positively related to porosity in vegetation concrete.

### Change laws for the water holding capacity and retention ability

**Figure 6:**
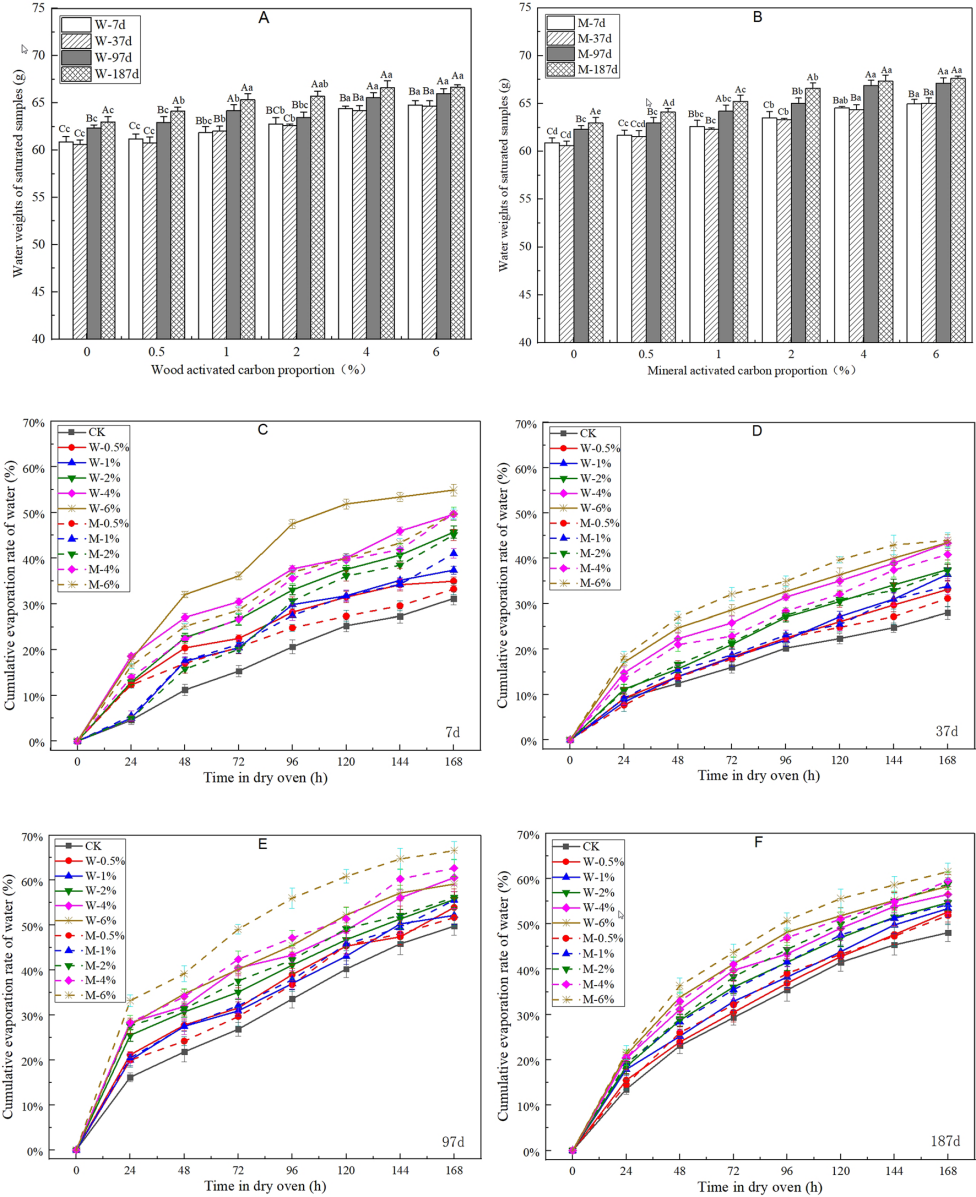
Effects of activated carbon on the water weight of saturated vegetation concrete (**A,B**) and cumulative evaporation rate of vegetation concrete (**C–F**) at different times. Different capital letters indicate significant differences between different times (*P* < 0.05). Different small letters indicate significant differences between different activated carbon proportions (*P* < 0.05).

Saturated water content can directly reflect the water holding capacity^25^, which represents the water supply capacity of vegetation concrete. As shown in Fig. 6A, B, the saturated water content increases with increasing proportion of activated carbon. With regard to samples containing wood activated carbon, the rate of increase is higher when the proportion increases from 0.5% to 1% than that in the other cases. In addition, the saturated water content of samples containing mineral activated carbon shows an insensitive change with time. In addition, the saturated water contents for samples containing wood activated carbon are lower than those for samples containing mineral activated carbon at 7 d. However, the opposite situation is observed at 187 d. Consequently, it can be deduced that the durability and potential for wood activated carbon may be better than that for mineral activated carbon.

The cumulative evaporation rate can reflect the water retention ability of vegetation concrete^26^. Figure 6C–F shows the curves for the cumulative evaporation rate of water with time. Although a positive relation between the water holding capacity and activated carbon proportion is found inFig. 6A, B, it can also be deduced that the water retention ability is negatively related to the activated carbon proportion according to Fig. 6C–F. The cumulative evaporation rates at 37 d are generally lower that those at other sampling times. This outcome may be mainly due to the fact that plant growth at 37 d is still in the seeding stage, at which point the leading factors affecting the pore structure of vegetation concrete are hydration reaction and consolidation, rather than plant roots. Thus, the connected pore volume decreases and the sealed pore volume increases, which can limit the water evaporation rate. After 37 d, plant roots grow gradually, and an increase in the connected pore volume can promote the water evaporation rate. Furthermore, curves obtained for samples containing activated carbon gradually move closer to those obtained for control samples from 37 to 187 d. This outcome indicates that the water retention ability of samples containing activated carbon can be enhanced with increasing time. With regard to the difference in the effects of the two activated carbon types, the cumulative evaporation rates for samples containing wood activated carbon are generally higher than those for mineral activated carbon at 7 d, while the opposite situation is observed at 187 d. It can be concluded that the water retention ability of wood activated carbon is better than that of mineral activated carbon over the long term.

### Change law for the shear strength

**Figure 7:**
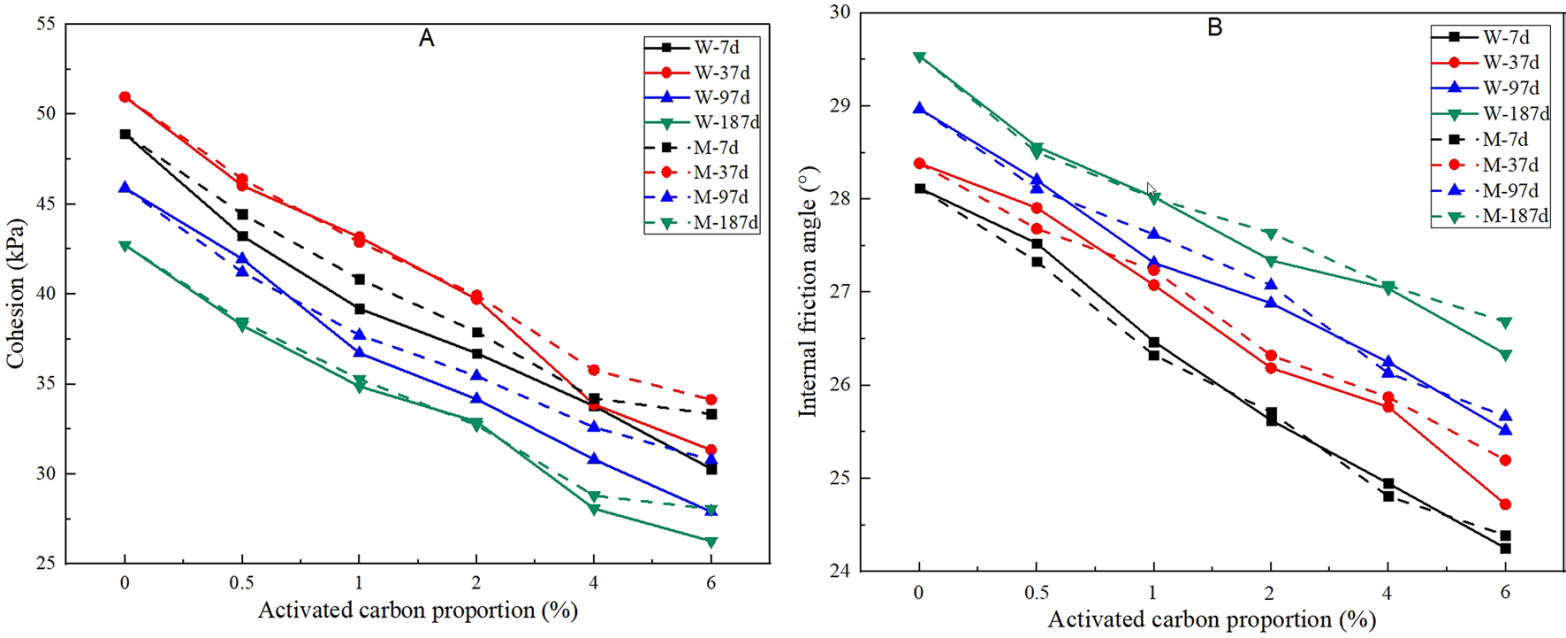
Effect of activated carbon on the cohesion (**A**) and internal friction angle (**B**) of vegetation concrete.

Shear strength, as reflected by the cohesive force and internal friction angle, is a very important factor for the anti-sliding ability of vegetation concrete on a slope^27^. Figure 7A, B shows the curves for the cohesive force and internal friction angle versus activated carbon proportion. It can be found that the two indexes both decrease with increasing proportion of activated carbon. As time goes on, the cohesive force increases first and then decreases. The reason for this outcome may be due to the fact that the whole hydration reaction often finishes in approximately one month^28^ and that the encapsulation effect between a soil particle and hydration products can enhance the cohesive force. However, there are large quantities of negative electrical charge present on the surface of activated carbon, and cations in the water film among the soil particles can be adsorbed^29^. Then, the thickness of the water film can increase, and the attraction among soil particles can be reduced naturally. The internal friction angle increases with increasing time, which may be because activated carbon mixing leads to changes in the arrangement of solid particles in the vegetation concrete, which can enhance the interlocking effect among solid particles^30^. In addition, the cohesive forces and internal friction angles for samples containing mineral activated carbon are both slightly higher than those for samples containing wood activated carbon.

### Change laws for nutrient content and retention ability

**Figure 8:**
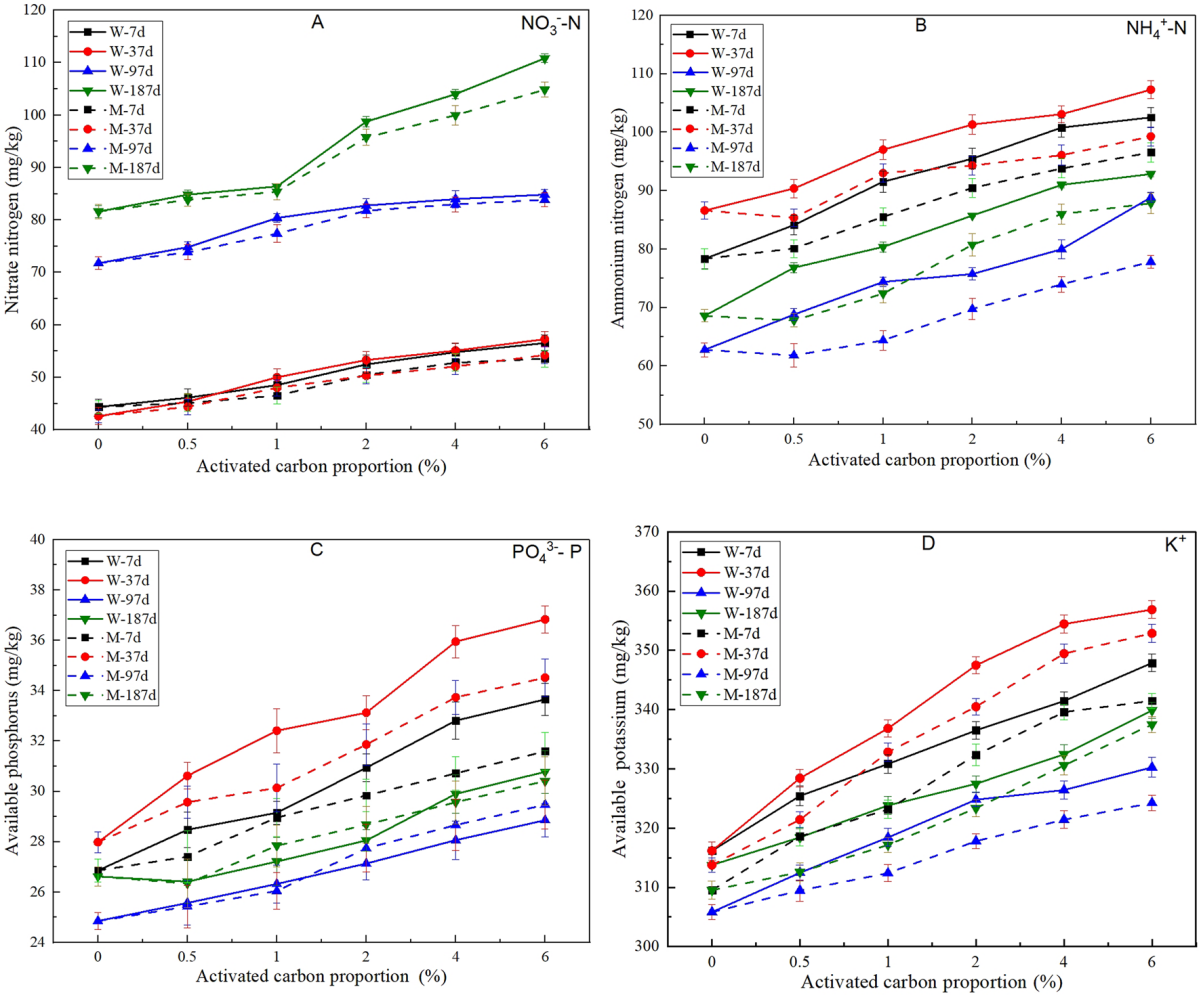
Effect of activated carbon on the contents of nitrate nitrogen (**A**), ammonium nitrogen (**B**), available phosphorus (**C**) and available potassium (**D**) in vegetation concrete.

Nutrient content of vegetation concrete is very important for plant growth^15^. Curves for nitrate nitrogen (NO_3_^−^–N), ammonium nitrogen (NH_4_^+^–N), available phosphorus (PO_4_^3−^–P) and potassium (K^+^) contents versus activated carbon proportion are shown in Fig. 8A–D. It can be found that the nutrient content of all types increases with increasing proportion of activated carbon, because there is a certain amount of nutrient in the activated carbon itself. In addition, there is no obvious change law for the ammonium nitrogen (NH_4_^+^–N), available phosphorus (PO_4_^3−^–P) and potassium (K^+^) content with increasing time, while the nitrate nitrogen (NO_3_^−^–N) increases with increasing time. In addition, the nutrient content in samples containing wood activated carbon is generally higher than that in samples containing mineral activated carbon. The main reason for this outcome may be that nutrient content in wood activated carbon is higher than that in mineral activated carbon, as shown in Table 2.

**Figure 9:**
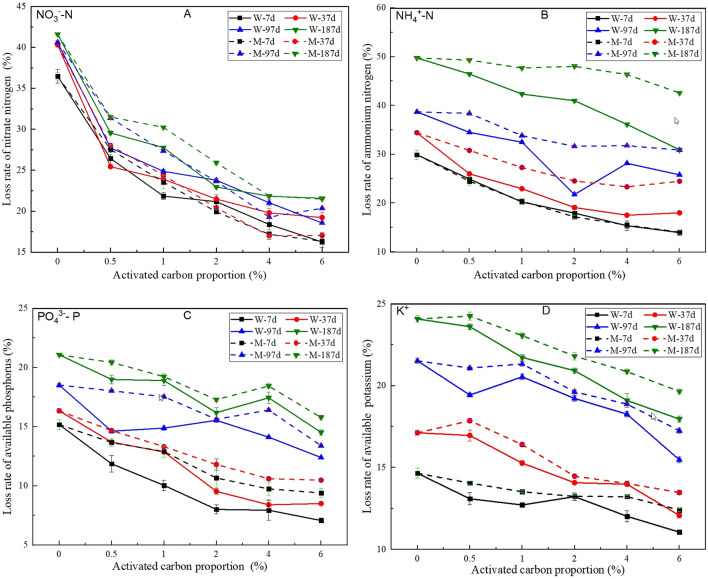
Effect of activated carbon on the loss rates of nitrate nitrogen (**A**), ammonium nitrogen (**B**), available phosphorus (**C**) and available potassium (**D**) of vegetation concrete.

The loss rate for nutrients, as measured by a leaching test, can directly reflect the nutrient retention ability, which represents the continuity of the nutrient supply^31^. According to a comparison of the data for samples containing activated carbon and control samples in Fig. 9A–D, it can be found that activated carbon can enhance the nutrient retention ability obviously and that the nutrient loss rate decreases with increasing proportion of activated carbon. Furthermore, the range of decrease in the loss rate of nitrate nitrogen (NO_3_^−^–N), ammonium nitrogen (NH_4_^+^–N) and available phosphorus (PO_4_^3−^–P) is obviously higher than that of available potassium (K^+^). The activated carbon surface, pyrolyzed under anaerobic conditions, is mainly occupied by negative electrical charges; thus, the adsorption ability for cations is commonly stronger than that for anions^32^. However, the activated carbon used in this research was modified by iron ions, and nitrate nitrogen (NO_3_^−^–N) and available phosphorus (PO_4_^3−^–P) can be adsorbed effectively due to hydrogen bonding and ligand exchange. Unlike ammonium nitrogen (NH_4_^+^–N), available potassium (K^+^) cannot react with the hydroxy (–OH) or carboxy (–COOH) groups located on the surface of the activated carbon. Therefore, a hydrogen bond cannot be formed, and, thus, the adsorption effect is weak. In summary, the adsorption mechanism for nitrate nitrogen (NO_3_^−^–N), ammonium nitrogen (NH_4_^+^–N) and available phosphorus (PO_4_^3−^–P) mainly belongs to chemical monolayer adsorption, while the adsorption mechanism for available potassium (K^+^) mainly belongs to physical multimolecular layer adsorption.

However, activated carbon can degrade gradually. According to Fig. 9A–D, the nutrient loss rates increase with increasing time, and the increasing range for ammonium nitrogen (NH_4_^+^–N) is clearly higher than that for the other three nutrient indexes. The reason for this outcome may be because the pH of vegetation concrete decreases with plant growth, resulting in an increase in the number of hydrogen ions (H^+^), which can weaken the adsorption ability for cations.

Moreover, the nutrient loss rates for samples containing wood activated carbon are generally lower than those for samples containing mineral activated carbon. It can be deduced that the nutrient retention ability of wood activated carbon is stronger than that of mineral activated carbon.

### Effect of activated carbon on plant

The average plant height, aboveground and underground biomass of *Cynodon dactylon* are shown in Table 4, respectively. The average plant height, aboveground biomass and underground biomass all increase as the activated carbon proportion increases from 0 to 2%, while the opposite trend is observed when the proportion exceeds 2%. This outcome shows that the higher proportion of activated carbon is not beneficial, although activated carbon is advantageous for plant growth. Furthermore, the average plant height and biomass of samples containing wood activated carbon is always higher than that of samples containing mineral activated carbon.

**Table 4:** Average plant height at different times and above/underground biomass at 187 d.

Sample type	Activated carbon proportion (%)	Average height of *Cynodon dactylon*/(cm)			Aboveground biomass at 187d/(g)	Underground biomass at 187d/(g)
		37d	97d	187d		
Control samples	0	22.97 ± 0.43Bc	23.29 ± 0.54ABc	24.15 ± 0.57Ac	12.38 ± 0.47c	4.85 ± 0.25c
Samples containing wood activated carbon	0.5	24.91 ± 0.54Bb	25.33 ± 0.37Bb	26.94 ± 0.50Ab	14.57 ± 0.46b	6.13 ± 0.28b
	1	26.86 ± 0.23Ba	27.58 ± 0.65Ba	29.33 ± 0.48Aa	14.97 ± 0.54ab	6.34 ± 0.34ab
	2	26.87 ± 0.46Ba	27.76 ± 0.47Ba	29.77 ± 0.39Aa	15.78 ± 0.71a	6.68 ± 0.25a
	4	25.47 ± 0.54Cb	27.33 ± 0.53Ba	29.25 ± 0.43Aa	15.23 ± 0.65ab	6.46 ± 0.22ab
	6	21.94 ± 0.39Cd	23.86 ± 0.61Bc	26.75 ± 0.67Ab	14.78 ± 0.84ab	6.09 ± 0.25b
Samples containing mineral activated carbon	0.5	23.42 ± 0.75Bab	24.92 ± 0.67Ab	25.35 ± 0.67Abc	14.16 ± 0.57b	5.56 ± 0.38b
	1	23.86 ± 0.68Bab	26.93 ± 0.75Aa	27.47 ± 0.71Aa	14.73 ± 0.62ab	5.98 ± 0.23ab
	2	24.97 ± 0.85Ba	27.42 ± 0.56Aa	28.13 ± 0.45Aa	15.53 ± 0.52a	6.24 ± 0.32a
	4	23.50 ± 0.65Bab	27.14 ± 0.73Aa	27.45 ± 0.51Aa	15.14 ± 0.65ab	6.19 ± 0.27ab
	6	21.31 ± 0.75Bc	24.33 ± 0.65Abc	25.25 ± 0.66Abc	14.15 ± 0.45b	6.03 ± 0.29ab

Different capital letters indicate significant differences between different times (*P* < 0.05). Different small letters indicate significant differences between different activated carbon proportions (*P* < 0.05).

### Microscopic images analysis

**Figure 10:**
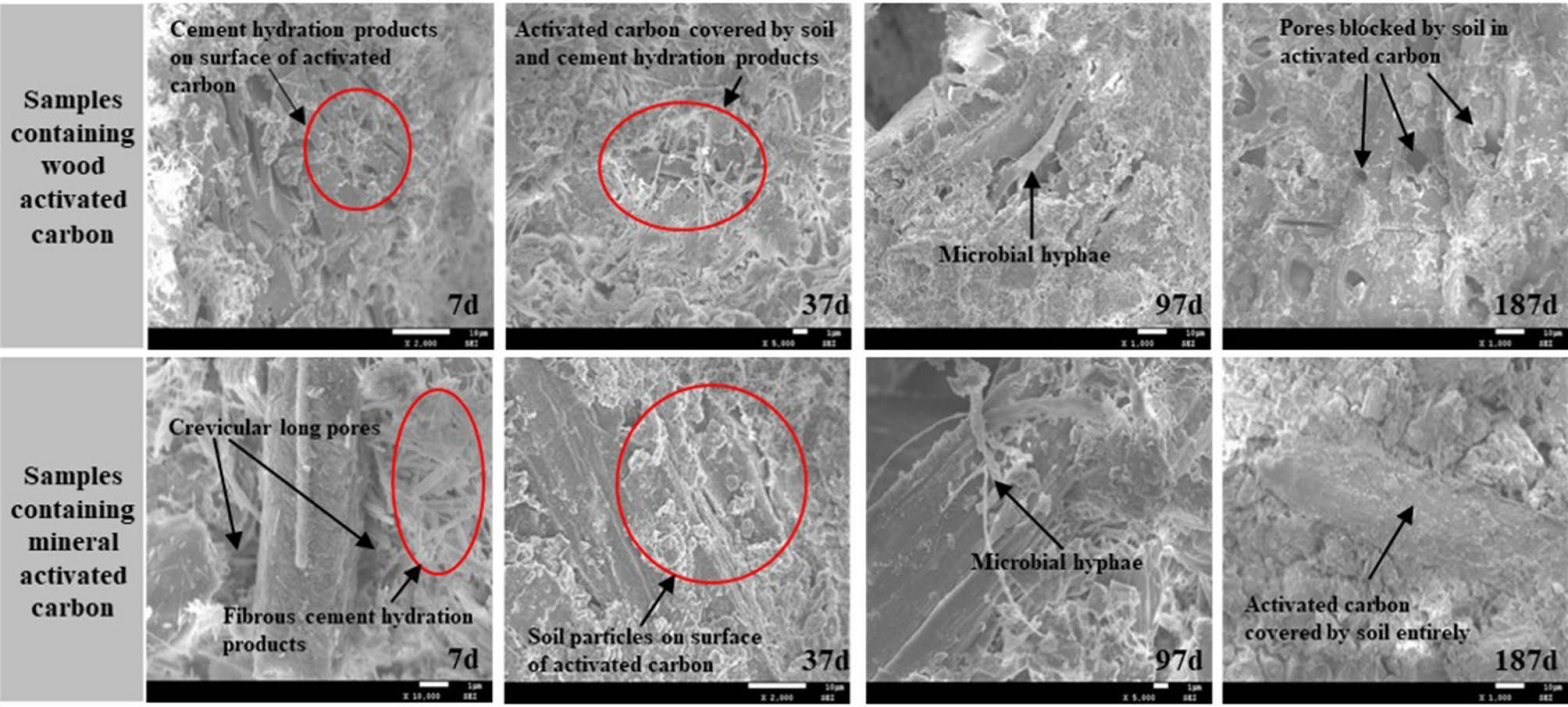
SEM images of samples taken at different times.

The microscopic images of vegetation concrete containing activated carbon at different times are shown in Fig. 10. From images taken at 7 d, many hydration products can be seen around the activated carbon, and some of the pores of wood activated carbon are even blocked by hydration products. However, the surface of both types of activated carbon is still smooth to some extent. According to images taken at 37 d, it can be found that both types of activated carbon are gradually covered by hydration products and soil. With completion of the main cement hydration reaction at 37 d, the microbes begin to reproduce rapidly. In addition, activated carbon with a porous structure can also provide suitable growing space and environment^33^; thus, the microbial quantity obviously increases and gathers together, which can promote the formation of microbial hyphae, as shown in images taken at 97 d^34^. As time goes on, the activated carbon is gradually covered by soil and hydration products, and the surface changes from being smooth to rough, as shown in the images taken at 187 d; in addition, with regard to the wood activated carbon, the pores also become blocked. As the adsorption ability of the activated carbon is mainly due to its large specific surface area and porous structure, Fig. 10 more clearly shows the ageing process for the activated carbon.

## Discussion

The porosity, dry density, shear strength, nutrient content are key evaluation indexes for the substrate in ecological slope protection engineering^35^. Based on the current Energy Industry Standard of the People’s Republic of China ‘*Technical Code for Eco-Restoration of Vegetation Concrete on Steep Slope of Hydropower Projects (NB/T 35082–2016*)’, a suitable porosity for vegetation concrete should be larger than 25%, and a suitable dry density should range between 1.10 and 1.47 g/cm^3^. In this test, the porosities and dry densities for almost all of the samples satisfy the above requirements expect for the treatment of W-6% at 187 d. The standard also stipulates that the fraction of available nitrogen, phosphorus and potassium should be larger than 60 mg/kg, 20 mg/kg and 100 mg/kg, respectively. The test results for all samples are in accordance with the specification. With regard to the shear strength, previous research has pointed out that the cohesive force and internal friction angle should be larger than 15 kPa and 30°, respectively, otherwise interfacial sliding between the vegetation concrete and slope can easily occur^36^. In this research, the cohesive forces for all samples were larger than 25 kPa, while the internal friction angles for most samples were smaller than 30°. As the thickness of vegetation concrete on a slope usually ranges between 8 to 12 cm, interfacial normal stress between vegetation concrete and slope produced by the self-weight of the vegetation concrete is always not large. Consequently, the interfacial shear strength is mainly due to the cohesive force according to the Mohr–Coulomb criterion^37^. This means that the shear strength of vegetation concrete still satisfies the standard requirement, although the internal friction angle is reduced by activated carbon mixing.

Although there are no obvious requirements for the indexes of permeability, water holding capacity and nutrient retention ability, they are still very important physicochemical indexes for plant growth. Based on the results from the experiments, it can be easily deduced that activated carbon mixing reduces the nutrient loss rate and promotes the permeability and water holding capacity, which are advantageous for plant growth. Moreover, the *Cynodon dactylon* growth situation, containing information about the plant height, aboveground and underground biomass, shows the beneficial effect of activated carbon on the vegetation concrete as well.

However, it is not true to admit that a larger proportion of activated carbon is better, such as the effect on the shear strength, plant height and biomass. This finding is somewhat similar to the research results reported by some scholars^38^. And it is important to note that the effect of wood activated carbon is generally better than that of mineral activated carbon, especially in terms of dry density, permeability, nutrient content and retention ability. In addition, with increasing time, the surface of activated carbon is covered by soil and cement hydration products, the pores in the activated carbon are also blocked by solid particles, and a part of the activated carbon is evenly dissolved in water and transformed into soluble carbon that can easily run off^39,40^. In other words, the activated carbon can deteriorate, as shown in Fig. 10. Based on the current standard, the mandatory curing period for vegetation concrete ecological protection engineering is always three months, while the working effect of activated carbon can last for at least half a year according to the results in this research. This finding means that activated carbon mixing can still effectively improve the properties of vegetation concrete in the early stage of the engineering service period, which is important for plant growth, and could decide the success or failure of the engineering.

Reasonable physicochemical properties of ecological substrates are essential conditions for the continuous healthy growth of plant. Existing engineering experience shows that there are some imperfections in normal vegetation concrete such as high density, low permeability, insufficient nutrient retention ability, and so on. However, a too loose structure can easily lead to an obvious decrease in mechanical property and nutrient retention ability^41^, which is disadvantageous for plant growth and the self-stability of vegetation on the slope. Therefore, activated carbon, of which the structure is porous and ionic adsorption ability is strong, was chosen to be mixed in vegetation concrete in this research. According to the research results, the effect of activated carbon can be summarized as follows: increased porosity, permeability, water holding capacity, nutrient content and retention ability; decreased dry density and shear strength. Furthermore, from analysis of SEM images, it can be deduced that the numerous micropore structures in the activated carbon can provide a habitat for microorganisms, which can enhance the nutrient levels for vegetation. By comprehensive consideration of the current industry standard, previous research results and economical reasoning, use of wood activated carbon is recommended, with a corresponding suitable proportion ranging between 1 and 2%. Under these conditions, the porosity, permeability, water holding capacity, and nutrient content and retention ability can reach a high level, and the shear strength can satisfy the self-stability requirement for vegetation concrete on a slope at the same time.

## Conclusion

The proportion of activated carbon in vegetation concrete is positively related to the porosity, permeability, water holding capacity, nutrient content and retention ability, while it is negatively related to the dry density, water retention ability, cohesive force and internal friction angle. When the proportion of activated carbon ranges between 0 to 2%, the plant height, aboveground and underground biomass of *Cynodon dactylon* all increase with increasing proportion of activated carbon, while the opposite trend is observed when the proportion ranges between 2 to 6%.

Generally, the effect of wood activated carbon is more remarkable than that of mineral activated carbon, especially in terms of dry density, permeability, nutrient content and retention ability, and the aboveground and underground biomass of the plant. With regard to shear strength, the performance of mineral activated carbon is slightly better than that of wood activated carbon.

From the perspective of plant growth requirement, the effects of activated carbon on porosity, dry density, water holding capacity, and nutrient content and retention ability are advantageous. From the perspective of the self-stability of vegetation concrete on a slope, the effects on the water retention ability and shear strength are disadvantageous. By comprehensive consideration of the current industry standard, previous research results and economical reasoning, the recommended type of activated carbon is wood, with a suitable proportion ranging between 1 and 2%.

Although activated carbon can slowly deteriorate with increasing time, it can still effectively improve the properties of vegetation concrete for at least half a year, which is important for the success of ecological protection engineering.

## Data Availability

The datasets generated during and/or analysed during the current study are available from the corresponding author upon reasonable request.
